# Supplementary material for: Braking performance oriented multi–objective optimal design of electro–mechanical brake parameters
Source: PLoS One. 2021 May 19;16(5):e0251714. doi: 10.1371/journal.pone.0251714 (PMC8133414; doi:10.1371/journal.pone.0251714)
Supplement: S1 Appendix — (DOCX) [file pone.0251714.s001.docx]

**S1 Appendix.** Orthogonal tests results.

| Tests | *R* | *L* | *M* | *p* | *ψ_m_* | *g* | *d_m_* | *l* | *J* | *D* | - | - | - | Response  time | Maximum  Braking  pressure |
| --- | --- | --- | --- | --- | --- | --- | --- | --- | --- | --- | --- | --- | --- | --- | --- |
| 1 | (1)  0.4 | (1)  0.0045 | (1)  0.0002 | (1)  3 | (1)  0.002 | (1)  3 | (1)  25 | (1)  4 | (1)  0.001 | (1)  0.001 | (1)  - | (1)  - | (1)  - | 0.57 | 68.81 |
| 2 | (1)  0.4 | (1)  0.0045 | (1)  0.0002 | (1)  3 | (2)  0.007 | (2)  4 | (2)  30 | (2)  5 | (2)  0.002 | (2)  0.002 | (2)  - | (2)  - | (2)  - | 0.83 | 122 |
| 3 | (1)  0.4 | (1)  0.0045 | (1)  0.0002 | (1)  3 | (3)  0.012 | (3)  5 | (3)  35 | (3)  6 | (3)  0.003 | (3)  0.003 | (3)  - | (3)  - | (3)  - | 0.88 | 121.9 |
| 4 | (1)  0.4 | (2)  0.0048 | (2)  0.00025 | (2)  5 | (1)  0.002 | (1)  3 | (1)  25 | (2)  5 | (2)  0.002 | (2)  0.002 | (3)  - | (3)  - | (3)  - | 0.66 | 118.63 |
| 5 | (1)  0.4 | (2)  0.0048 | (2)  0.00025 | (2)  5 | (2)  0.007 | (2)  4 | (2)  30 | (3)  6 | (3)  0.003 | (3)  0.003 | (1)  - | (1)  - | (1)  - | 0.56 | 121.19 |
| 6 | (1)  0.4 | (2)  0.0048 | (2)  0.00025 | (2)  5 | (3)  0.012 | (3)  5 | (3)  35 | (1)  4 | (1)  0.001 | (1)  0.001 | (2)  - | (2)  - | (2)  - | 0.89 | 121.03 |
| 7 | (1)  0.4 | (3)  0.005 | (3)  0.0003 | (3)  8 | (1)  0.002 | (1)  3 | (1)  25 | (3)  6 | (3)  0.003 | (3)  0.003 | (2)  - | (2)  - | (2)  - | 0.57 | 122.02 |
| 8 | (1)  0.4 | (3)  0.005 | (3)  0.0003 | (3)  8 | (2)  0.007 | (2)  4 | (2)  30 | (1)  4 | (1)  0.001 | (1)  0.001 | (3)  - | (3)  - | (3)  - | 0.75 | 121.04 |
| 9 | (1)  0.4 | (3)  0.005 | (3)  0.0003 | (3)  8 | (3)  0.012 | (3)  5 | (3)  35 | (2)  5 | (2)  0.002 | (2)  0.002 | (1)  - | (1)  - | (1)  - | 0.91 | 122.02 |
| 10 | (2)  0.5 | (1)  0.0045 | (2)  0.00025 | (3)  8 | (1)  0.002 | (2)  4 | (3)  35 | (1)  4 | (2)  0.002 | (3)  0.003 | (1)  - | (2)  - | (3)  - | 0.87 | 122.01 |
| 11 | (2)  0.5 | (1)  0.0045 | (2)  0.00025 | (3)  8 | (2)  0.007 | (3)  5 | (1)  25 | (2)  5 | (3)  0.003 | (1)  0.001 | (2)  - | (3)  - | (1)  - | 0.74 | 121.1 |
| 12 | (2)  0.5 | (1)  0.0045 | (2)  0.00025 | (3)  8 | (3)  0.012 | (1)  3 | (2)  30 | (3)  6 | (1)  0.001 | (2)  0.002 | (3)  - | (1)  - | (2)  - | 0.5 | 121.02 |
| 13 | (2)  0.5 | (2)  0.0048 | (3)  0.0003 | (1)  3 | (1)  0.002 | (2)  4 | (3)  35 | (2)  5 | (3)  0.003 | (1)  0.001 | (3)  - | (1)  - | (2)  - | 0.94 | 79.5 |
| 14 | (2)  0.5 | (2)  0.0048 | (3)  0.0003 | (1)  3 | (2)  0.007 | (3)  5 | (1)  25 | (3)  6 | (1)  0.001 | (2)  0.002 | (1)  - | (2)  - | (3)  - | 0.65 | 121.04 |
| 15 | (2)  0.5 | (2)  0.0048 | (3)  0.0003 | (1)  3 | (3)  0.012 | (1)  3 | (2)  30 | (1)  4 | (2)  0.002 | (3)  0.003 | (2)  - | (3)  - | (1)  - | 0.61 | 121.12 |
| 16 | (2)  0.5 | (3)  0.005 | (1)  0.0002 | (2)  5 | (1)  0.002 | (2)  4 | (3)  35 | (3)  6 | (1)  0.001 | (2)  0.002 | (2)  - | (3)  - | (1)  - | 0.59 | 96.67 |
| 17 | (2)  0.5 | (3)  0.005 | (1)  0.0002 | (2)  5 | (2)  0.007 | (3)  5 | (1)  25 | (1)  4 | (2)  0.002 | (3)  0.003 | (3)  - | (1)  - | (2)  - | 0.85 | 121.02 |
| 18 | (2)  0.5 | (3)  0.005 | (1)  0.0002 | (2)  5 | (3)  0.012 | (1)  3 | (2)  30 | (2)  5 | (3)  0.003 | (1)  0.001 | (1)  - | (2)  - | (3)  - | 0.52 | 121.01 |
| 19 | (3)  0.6 | (1)  0.0045 | (3)  0.0003 | (2)  5 | (1)  0.002 | (3)  5 | (2)  30 | (1)  4 | (3)  0.003 | (2)  0.002 | (1)  - | (3)  - | (2)  - | 1.21 | 124.05 |
| 20 | (3)  0.6 | (1)  0.0045 | (3)  0.0003 | (2)  5 | (2)  0.007 | (1)  3 | (3)  35 | (2)  5 | (1)  0.001 | (3)  0.003 | (2)  - | (1)  - | (3)  - | 0.55 | 121.02 |
| 21 | (3)  0.6 | (1)  0.0045 | (3)  0.0003 | (2)  5 | (3)  0.012 | (2)  4 | (1)  25 | (3)  6 | (2)  0.002 | (1)  0.001 | (3)  - | (2)  - | (1)  - | 0.56 | 121.04 |
| 22 | (3)  0.6 | (2)  0.0048 | (1)  0.0002 | (3)  8 | (1)  0.002 | (3)  5 | (2)  30 | (2)  5 | (1)  0.001 | (3)  0.003 | (3)  - | (2)  - | (1)  - | 0.92 | 122.01 |
| 23 | (3)  0.6 | (2)  0.0048 | (1)  0.0002 | (3)  8 | (2)  0.007 | (1)  3 | (3)  35 | (3)  6 | (2)  0.002 | (1)  0.001 | (1)  - | (3)  - | (2)  - | 0.47 | 121.04 |
| 24 | (3)  0.6 | (2)  0.0048 | (1)  0.0002 | (3)  8 | (3)  0.012 | (2)  4 | (1)  25 | (1)  4 | (3)  0.003 | (2)  0.002 | (2)  - | (1)  - | (3)  - | 1.01 | 123.03 |
| 25 | (3)  0.6 | (3)  0.005 | (2)  0.00025 | (1)  3 | (1)  0.002 | (3)  5 | (2)  30 | (3)  6 | (2)  0.002 | (1)  0.001 | (2)  - | (1)  - | (3)  - | 0.89 | 109.3 |
| 26 | (3)  0.6 | (3)  0.005 | (2)  0.00025 | (1)  3 | (2)  0.007 | (1)  3 | (3)  35 | (1)  4 | (3)  0.003 | (2)  0.002 | (3)  - | (2)  - | (1)  - | 0.73 | 122.02 |
| 27 | (3)  0.6 | (3)  0.005 | (2)  0.00025 | (1)  3 | (3)  0.012 | (2)  4 | (1)  25 | (2)  5 | (1)  0.001 | (3)  0.003 | (1)  - | (3)  - | (2)  - | 0.67 | 121.12 |
