# Supplementary material for: Braking performance oriented multi–objective optimal design of electro–mechanical brake parameters
Source: PLoS One. 2021 May 19;16(5):e0251714. doi: 10.1371/journal.pone.0251714 (PMC8133414; doi:10.1371/journal.pone.0251714)
Supplement: S2 Appendix — (DOCX) [file pone.0251714.s002.docx]

**S2 Appendix.** Range analyses.

| Indexes | | *R* | *L* | *M* | *p* | *ψ_m_* | *g* | *d_m_* | *l* | *J* | *D* |
| --- | --- | --- | --- | --- | --- | --- | --- | --- | --- | --- | --- |
| $K_{1}$ | Response time | 6.615 | 6.705 | 6.633 | 6.768 | 6.273 | 5.175 | 6.273 | 7.488 | 6.084 | 6.327 |
|  | Max Braking pressure | 1038.64 | 1042.95 | 1017.49 | 986.81 | 963 | 1036.69 | 1037.81 | 1044.13 | 1013.76 | 983.87 |
| $K_{2}$ | Response time | 6.273 | 6.714 | 6.507 | 6.39 | 6.129 | 6.84 | 6.786 | 6.957 | 6.651 | 7.092 |
|  | Max Braking pressure | 1024.49 | 1048.59 | 1077.42 | 1065.66 | 1091.47 | 1027.6 | 1082.74 | 1048.41 | 1078.18 | 1070.48 |
| $K_{3}$ | Response time | 7.011 | 6.48 | 6.75 | 6.741 | 6.552 | 7.938 | 6.831 | 5.67 | 7.29 | 6.48 |
|  | Max Braking pressure | 1084.63 | 1056.22 | 1052.85 | 1095.29 | 1093.29 | 1083.47 | 1027.21 | 1055.22 | 1055.82 | 1093.41 |
| $\overline{K}_{1}$ | Response time | 0.735 | 0.745 | 0.737 | 0.752 | 0.697 | 0.575 | 0.697 | 0.832 | 0.676 | 0.703 |
|  | Max Braking pressure | 115.4 | 115.88 | 113.05 | 109.65 | 107 | 115.19 | 115.31 | 116.01 | 112.64 | 109.32 |
| $\overline{K}_{2}$ | Response time | 0.697 | 0.746 | 0.723 | 0.71 | 0.681 | 0.76 | 0.754 | 0.773 | 0.739 | 0.788 |
|  | Max Braking pressure | 113.83 | 116.51 | 119.71 | 118.41 | 121.27 | 114.18 | 120.3 | 116.49 | 119.8 | 118.94 |
| $\overline{K}_{3}$ | Response time | 0.779 | 0.72 | 0.75 | 0.749 | 0.728 | 0.882 | 0.759 | 0.63 | 0.81 | 0.72 |
|  | Max Braking pressure | 120.51 | 117.36 | 116.98 | 121.7 | 121.48 | 120.39 | 114.13 | 117.25 | 117.31 | 121.49 |
| $R$ | Response time | 0.082 | 0.026 | 0.027 | 0.042 | 0.047 | 0.307 | 0.062 | 0.202 | 0.134 | 0.068 |
|  | Max Braking pressure | 6.68 | 1.48 | 6.66 | 12.05 | 14.48 | 6.21 | 6.17 | 1.24 | 7.16 | 12.17 |
