# Supplementary material for: Braking performance oriented multi–objective optimal design of electro–mechanical brake parameters
Source: PLoS One. 2021 May 19;16(5):e0251714. doi: 10.1371/journal.pone.0251714 (PMC8133414; doi:10.1371/journal.pone.0251714)
Supplement: Attachment — Submitted filename: PONE-D-20-39894 comments.pdf [file pone.0251714.s003.pdf]

PLOS ONE journal

Manuscript Number: PONE-D-20-39894

Title: Braking performance oriented multi–objective optimal design of electro–mechanical brake parameters

Comments and Suggestions for Authors

The manuscript entitled “Braking performance oriented multi–objective optimal design of electro–mechanical.”

This study is to provide a new idea for the parameters optimization of vehicle braking systems. In this way, the influence of the load transfer, the braking distribution, the ABS effects and the parameters coupling on the vehicle’s braking performance can be reduced, and the vehicle performance can be optimized. Therefore, the following work was conducted. the presented results of this work could encourage the overall uses of multivariate methods in these fields. All presented results are well discussed. It is also easy to understand. also, the paper structure is suitable and well written. In addition, the figures and table are clear and useful. Also, this paper is interesting and well written.

I encourage the journal to accept it with minor corrections.

1. The authors should indicate the possible impact of their work. Also need to add the novelty and main contributions clearly. I encourage the authors to add problem statement in the abstract or introduction.

2. The authors should discuss the problem statement and the novelty clearly in the introduction part.

3. The authors should be revised the conclusion with proper key results.

4. Figures 4, 5, 9 and 10 are not clear should be enhance.

5. I suggest adding your main contributions comparing with other researchers clearly (what is the difference with other researchers) what is the new in your study... hope you could add it in your paper.

6. I suggest adding future recommendations to the next researchers after the conclusion part.
